# Clinical Characteristics and Prognosis of Primary Central Nervous System Lymphoma: A Retrospective Analysis

**DOI:** 10.3390/cancers18030541

**Published:** 2026-02-06

**Authors:** Shupeng Zhong, Linjun Zhao, Jin Chai, Lan Mi, Yan Xie, Lingyan Ping, Xiaopei Wang, Jun Zhu, Lijuan Deng, Yuqin Song

**Affiliations:** 1 Key Laboratory of Carcinogenesis and Translational Research (Ministry of Education/Beijing), Department of Lymphoma, Peking University Cancer Hospital & Institute, Beijing 100142, China; zhongshupeng@163.com (S.Z.); 2411110734@bjmu.edu.cn (J.C.); milan@bjmu.edu.cn (L.M.); yanxie@bjmu.edu.cn (Y.X.); dingdingply1981@pku.edu.cn (L.P.); wangxiaopei@pkuih.edu.cn (X.W.); zhujun@csco.org.cn (J.Z.); 2 Lymphoma Unit, Peking University International Hospital, Beijing 102200, China; zhaolinjun@pkuih.edu.cn

**Keywords:** primary central nervous system lymphoma, induction therapy, consolidation therapy, survival analysis

## Abstract

Background: Primary central nervous system lymphoma (PCNSL) is a rare extranodal lymphoma characterized by a poor prognosis due to high relapse rates and a lack of standardized treatment. This study aimed to evaluate the impact of induction/consolidation therapy on long-term survival and to provide extended follow-up data. Methods: In this retrospective analysis, 140 immunocompetent patients with diffuse large B-cell PCNSL (DLBCL-PCNSL) treated at two centers between 2014 and 2024 were enrolled. Treatment efficacy was assessed based on baseline characteristics, therapeutic regimens, and treatment response. Progression-free survival (PFS) and overall survival (OS) were estimated using the Kaplan–Meier method, and prognostic factors were identified using multivariate Cox proportional hazards regression models. Results: With a median follow-up of 5.3 years (range: 0.1–11.0 years), the 2- and 5-year PFS rates were 50.4% (95% CI: 42.1–60.2) and 34.1% (95% CI: 25.5–45.0), respectively, while the corresponding OS rates were 85.3% (95% CI: 79.4–91.6) and 60.8% (95% CI: 52.0–71.1). No survival plateau was observed. Among patients, 94% received methotrexate-based induction therapy: 94 received rituximab–methotrexate–temozolomide (R-MT) and 17 received MT alone, with 2-year PFS rates of 57.7% and 39.7%, respectively. Overall, 75% of patients achieved remission (CR/CRu/PR) after induction, and among these, 55% underwent consolidation therapy, predominantly autologous stem cell transplantation (ASCT, 90%) or whole-brain radiotherapy (10%). Patients receiving ASCT exhibited superior survival outcomes compared to those who did not. Conclusions: R-MT induction combined with ASCT consolidation is associated with improved survival in PCNSL, although relapse risk remains substantial. Outcomes remain poor in refractory subgroups, highlighting the need for novel therapeutic strategies.

## Simple Summary

Primary central nervous system lymphoma (PCNSL) is a rare extranodal lymphoma with poor prognosis due to high relapse rates and a lack of standardized treatment. This retrospective analysis of 140 immunocompetent diffuse large B-cell PCNSL patients (treated between 2014 and 2024) shows that methotrexate-based induction therapy (notably the rituximab–methotrexate–temozolomide (R-MT) regimen) achieved 75% remission, and consolidation therapy (predominantly autologous stem cell transplantation) improved survival: 5-year overall survival reached 60.8% after a median 5.3-year follow-up. No survival plateau was observed, highlighting the need to standardize PCNSL treatment strategies.

## 1. Introduction

Primary central nervous system lymphoma (PCNSL) is a rare, aggressive non-Hodgkin lymphoma, confined to the central nervous system (CNS), accounting for 4–6% of extra-nodal lymphomas [1]. Over 90% exhibit diffuse large B-cell pathology [2], localized to immune-privileged regions, posing treatment challenges and high recurrence risk [3].

Standard management involves induction and consolidation therapy. MTX-based treatment is generally regarded as the standard induction therapy, though the optimal approach is undefined, and rituximab’s role remains debated [4]. Consolidation options encompass ASCT, non-myeloablative chemotherapy, and radiotherapy [5]. Although the IELSG-32 trial demonstrated the long-term efficacy of the MATRix regimen (HD-MTX, cytarabine, thiotepa, and rituximab) followed by ASCT consolidation [6], the application of such high-intensity regimens is often limited by severe hematological toxicity, particularly in elderly patients or those with poor performance status [7]. In Asian populations, evidence regarding the optimal therapeutic strategy for PCNSL remains fragmented. Although the R-MT regimen (rituximab, methotrexate, and temozolomide) is widely adopted due to its favorable tolerability profile, previous studies evaluating its efficacy have been constrained by small sample sizes [8]. While larger Asian cohort studies have emerged recently, they are often limited by short follow-up durations (median follow-up < 3 years) or substantial heterogeneity in treatment protocols, making it difficult to robustly assess long-term survival outcomes [9,10].

The median survival duration of untreated PCNSL patients is merely three months [11]. For multidrug combination chemotherapy based on HD-MTX, median progression-free (mPFS) and overall (mOS) survival durations of 6–24 and 14–66 months have been reported, respectively [12,13,14]. PCNSL demonstrates significant sensitivity to chemotherapy and radiotherapy, exhibiting an initial overall response rate (ORR) of roughly 70–80%; however, remission tends to be transient. Among patients who attain complete remission following induction chemotherapy, approximately 50% experience relapse, with older patients exhibiting an earlier onset of relapse, underscoring the critical need for effective yet tolerable consolidation strategies [15].

To bridge the gap in large-scale data for Asian populations and optimize the therapeutic balance between efficacy and safety, this study analyzed a large dual-center Chinese cohort of 140 patients. We provided extended follow-up data to evaluate the efficacy of induction treatment primarily based on R-MT and consolidation treatment based on autologous transplantation in newly diagnosed PCNSL patients, while also exploring high-risk factors associated with disease recurrence and progression.

## 2. Materials and Methods

### 2.1. Cohort Inclusion Criteria

This was a retrospective clinical study carried out at Peking University Cancer Hospital and Peking University International Hospital in China. Immunocompetent PCNSL cases diagnosed from 2014 to 2024 were selected for this study, which were confirmed as diffuse large B-cell lymphoma (DLBCL) through pathological examination of brain tumor biopsy specimens in accordance with the criteria outlined in the 4th edition of the WHO classification of tumors of haematopoietic and lymphoid tissues published in 2008 [16]. Exclusion criteria comprised non-diffuse large B-cell lymphoma pathology, indications of systemic disease beyond the CNS at initial presentation (i.e., secondary CNSL), insufficient treatment information (including treatment duration and specific regimen), and absence of ≥1 imaging evaluation (CT or MRI) following the initiation of treatment. The demographic characteristics, International Extranodal Lymphoma Study Group (IELSG) prognostic index, and Memorial Sloan Kettering Cancer Center (MSKCC) classification of the included patients were recorded.

### 2.2. Data Collection and Quality Control

Both participating centers, Peking University Cancer Hospital and Peking University International Hospital, adhered to identical clinical protocols and treatment regimens. Retrospective data were systematically extracted from electronic medical records based on pre-defined variables. Critical data points, particularly those pertaining to treatment response and survival outcomes, underwent rigorous validation to ensure accuracy and consistency.

### 2.3. Characteristics of the Primary Disease and Its Management

The baseline lesion distribution, ocular symptoms, meningeal involvement, and other conditions were recorded when available. The details of the first-line induction therapy regimen were thoroughly documented, while the first-line consolidation regimen encompassed autologous transplantation and radiotherapy, with additional specifics on the autologous transplantation conditioning regimen gathered. Patients exhibiting progressive (PD) or stable (SD) disease upon induction treatment were categorized as primary refractory cases. Patients exhibiting disease progression prior to consolidation therapy had each subsequent treatment categorized as salvage therapy, rather than consolidation therapy. Surgical cytoreduction exceeding the limits of the diagnostic biopsy was categorized as resection.

MT ± R regimen: For the core methotrexate (MTX) dosing: Patients aged ≤65 years received high-dose MTX (HD-MTX) at 3.5 g/m^2^ via intravenous infusion on Day 1, administered once every 2 weeks with a 4-week period as one cycle, and 3 cycles were planned. Patients aged >65 years received HD-MTX at a dose of 1.0–3.5 g/m^2^ via intravenous infusion on Day 1, with a 3-week period as one cycle, and 6 cycles were planned. All patients were concurrently administered temozolomide (TMZ) at 150 mg/m^2^ orally on Days 1–5 per cycle; rituximab at 375 mg/m^2^ (intravenous infusion on Day 0) could be added or not based on disease status.

### 2.4. Imaging Assessment

Diagnostic imaging primarily relied on contrast-enhanced magnetic resonance imaging (MRI) of the brain, interpreted by experienced neuroradiologists. Treatment response was evaluated in accordance with the International Primary Central Nervous System Lymphoma Collaborative Group (IPCG) consensus guidelines. Response assessments were performed at mid-induction, after the completion of induction therapy, after consolidation therapy, and every 3 months during surveillance (or immediately upon clinical suspicion of progression). Baseline systemic staging was performed using 18F-FDG PET/CT or contrast-enhanced CT of the chest, abdomen, and pelvis to exclude extra-CNS lymphoma involvement [17].

### 2.5. Statistical Analysis

This research employed Kaplan–Meier curves and the Cox proportional hazards model for assessing OS and PFS in patients. OS was determined from treatment initiation to death for any reason, loss to follow-up, or study conclusion. PFS was assessed from treatment commencement to disease progression, death, loss to follow-up, or the study’s conclusion. OS and PFS were estimated using the Kaplan–Meier method, and 95% confidence intervals (CIs) were calculated using the Greenwood formula. Simultaneously, the log-rank test was applied to ascertain whether the disparities in survival curves among various groups are statistically significant. Data completeness for critical baseline variables is summarized in Supplementary Table S3. For variables with missing data, a complete-case analysis approach was utilized, where cases with missing values were excluded from the specific hypothesis testing associated with that variable to ensure the accuracy of results based on observed clinical data.

To identify independent prognostic risk factors for PCNSL patients, we initially conducted univariate Cox regression analysis to screen potential prognostic variables (selection criterion: *p* < 0.1). Variables meeting this threshold were subsequently included in a multivariate Cox regression model to adjust for confounding factors. The proportional hazards assumption was verified using scaled Schoenfeld residuals. The significance of individual covariates in the Cox models was assessed using the Wald test. The final model incorporated the Memorial Sloan Kettering Cancer Center (MSKCC) risk score, deep brain structure involvement, treatment regimens, and other relevant prognostic factors, clarifying the independent effects of these factors on patient outcomes and providing more reliable references for clinical treatment decisions and prognostic assessment.

## 3. Results

### 3.1. Baseline Patient Characteristics and Treatment Modalities

**Figure 1:**
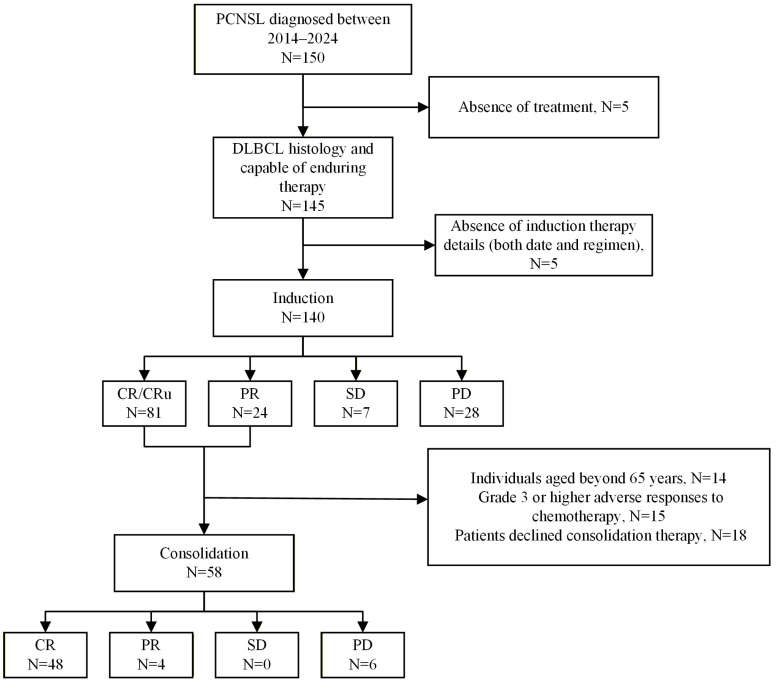
Study flowchart. PCNSL, primary central nervous system lymphoma; DLBCL, diffuse large B-cell lymphoma. CR/CRu, complete response/unconfirmed complete response; PR, partial response; SD, stable disease; PD, progressive disease. Study flowchart. PCNSL, primary central nervous system lymphoma; DLBCL, diffuse large B-cell lymphoma. CR/CRu, complete response/unconfirmed complete response; PR, partial response; SD, stable disease; PD, progressive disease.

Out of 150 individuals diagnosed with PCNSL from 2014 to 2024, 5 were excluded due to poor general condition at initial diagnosis and failure to receive treatment, and another 5 were excluded due to incomplete treatment information, including treatment duration and specific regimens. Ultimately, 140 patients were included, who had adequate treatment data (Figure 1).

Approximately 50% of the patients presented with a single lesion at the initial diagnosis (69/136, 51%). Most cases were classified as high-risk based on the MSKCC prognostic model (64/140, 46%), and the IELSG prognostic index ranged from 2 to 3 points (50/73, 68%). A limited proportion of patients exhibited combined ocular symptoms (33/140, 24%) or meningeal involvement (6/70, 9%) at the initial diagnosis (Table 1).

**Figure 2:**
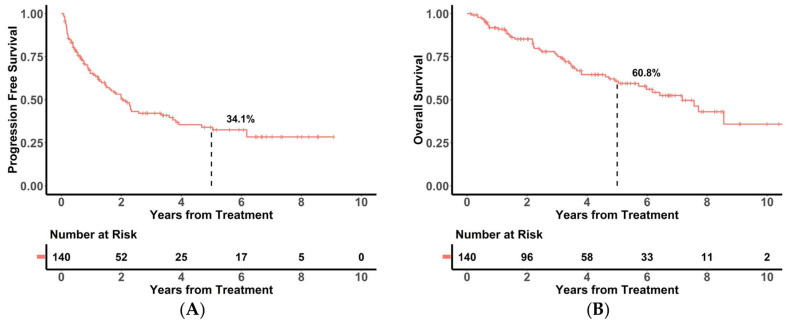
Kaplan–Meier analysis of (**A**) PFS and (**B**) OS in all patients.

Median patient age at diagnosis was 57 years (16–79 years), with a median follow-up duration of 5.3 years (range: 0.1–11.0 years). Median PFS was 2.1 years (95% CI: 1.5–2.6 years), and median OS was 7.2 years (95% CI: 5.7–8.7 years). The 5-year PFS rate was 34.1% (95% CI: 25.5–45.0%), and the 5-year OS rate was 60.8% (95% CI: 52.0–71.1%) (Figure 2).

A majority of patients (131, 94%) were administered methotrexate-based therapy, while a significant proportion (114/140, 81%) received rituximab-containing therapy. The predominant induction regimen was rituximab, methotrexate, and temozolomide (R-MT; 94/140, 67%), followed by methotrexate and temozolomide (MT; 17/140, 12%). Less frequently used approaches comprised R-MTX (7/140, 5%), Bruton’s tyrosine kinase (BTK) inhibitor-containing regimens (6/140, 4%), MTX (4/140, 3%), and R-T-thiotepa (2/140, 1%), among others. Alongside systemic induction therapy, the majority of patients (111/127, 87%) received intrathecal therapy, while a subset of cases underwent surgical resection at diagnosis (47/140, 34%) (Table 1).

### 3.2. Efficacy of Induction Therapy and Survival Analysis

No treatment-related deaths were observed during induction therapy (Table S4). Only 4% (5/140) of the patients required treatment regimen adjustments due to adverse reactions triggered by induction therapy. Following induction therapy, 58% (81/140) of patients achieved CR/CRu (including 77 cases of CR and 4 cases of CRu), 17% (24/140) attained PR, 5% (7/140) exhibited SD, and 20% (28/140) were classified as PD (Figure 1).

**Figure 3:**
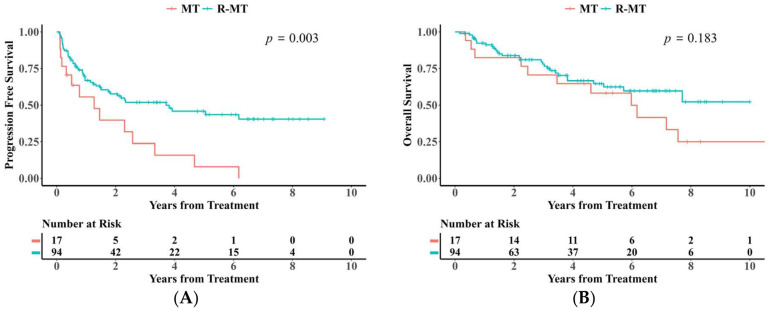
(**A**) Kaplan–Meier analysis of PFS between MT and R-MT groups. (**B**) Kaplan–Meier analysis of OS between MT and R-MT groups.

PFS was markedly improved in patients administered the R-MT regimen versus cases who received the MT regimen (log-rank, *p* < 0.05, Figure 3A). Additionally, OS between the two groups gradually widened after 6 years of treatment (Figure 3B). After 1:1 propensity score matching (PSM) for age, gender, and MSKCC score, the log-rank test still showed a statistically significant difference in PFS between the two groups (caliper value = 0.02, *p* < 0.05).

**Figure 4:**
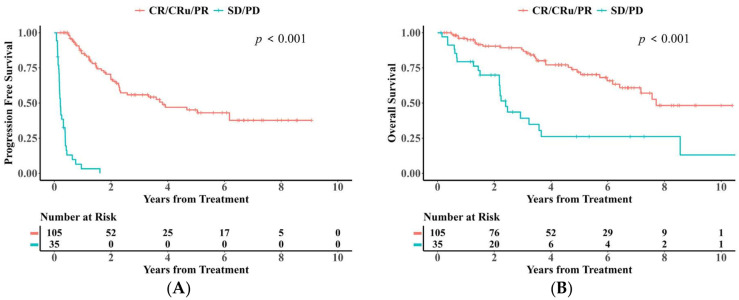
(**A**) Kaplan–Meier analysis of PFS stratified by induction therapy efficacy. (**B**) Kaplan–Meier analysis of OS stratified by induction therapy efficacy.

Patients who achieved a response (CR/CRu/PR) after induction therapy demonstrated significantly better survival compared to those with refractory disease (SD/PD) (Figure 4A,B). For responders, the 2-year and 5-year PFS rates were 66.7% (95% CI: 57.3–77.5%) and 45.1% (95% CI: 34.8–58.5%), respectively, while the corresponding OS rates were 90.5% (95% CI: 84.7–96.6) and 72.0% (95% CI: 62.5–83.0%). In contrast, the refractory group had a median PFS of only 0.2 years (95% CI: 0.2–0.3 years) and a median OS of 2.4 years (95% CI: 2.0–2.8 years) (Figure 4A,B and Figure S1A,B).

Regarding the impact of age on survival, patients aged <65 years had a higher 5-year PFS than those aged ≥65 years (36.2% [95% CI: 27.1–48.4%] vs. 26.6% [95% CI: 12.4–57.1%]), although this difference did not reach statistical significance (*p* = 0.499). Similarly, while the OS advantage for younger patients became increasingly pronounced over time, the difference in OS was not statistically significant (*p* = 0.161) (Figure S4A,B).

### 3.3. Efficacy of Consolidation Treatment and Survival Analysis

Of the 105 cases responding to induction therapy (evaluated as CR/CRu/PR), 55% (58/105) received consolidation therapy, of whom 90% (52/58) received ASCT consolidation. Among cases not administered consolidation therapy, 35 had poor treatment response or disease progression, 14 did not receive consolidation therapy due to age above 65 years, 15 had ≥grade 3 adverse reactions during induction therapy, and 18 refused subsequent consolidation therapy. Among the patients who received ASCT, only 1 patient was older than 65 years. Treatment efficacy in patients who received consolidation therapy was evaluated as follows: CR, N = 48; PR, N = 4; SD, N = 0; PD, N = 6 (Figure 1). Among the patients administered ASCT as consolidation therapy, 37% (19/52) received thiotepa-based conditioning regimens, 29% (15/52) received CBV regimens, 13% (7/52) received BEAC regimens, 8% (4/52) received BEAM regimens, and the remaining patients were treated with other regimens such as CFV, FEAC, or TB (Supplementary Table S1).

**Figure 5:**
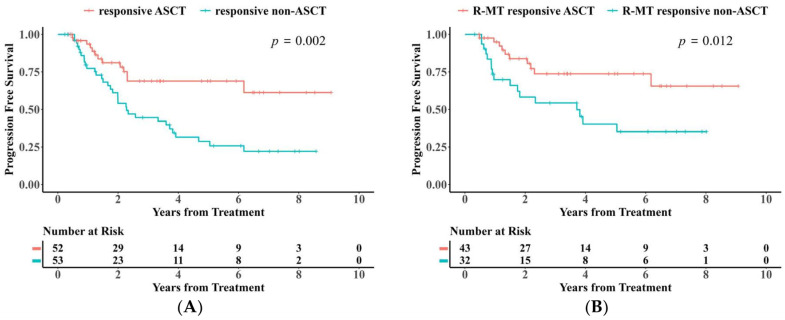
(**A**) Kaplan–Meier analysis of PFS between ASCT-receiving and non-ASCT-receiving groups among patients with responsive induction therapy. (**B**) Kaplan–Meier Analysis of PFS between ASCT-receiving and non-ASCT-receiving subgroups among patients with responsive R-MT induction therapy.

Following consolidation therapy, the majority of patients achieved CR (48/58, 83%). Among the 12 patients with PR after induction therapy who proceeded to ASCT consolidation, 9 subsequently attained CR (9/12, 75%) (Figure 1). Notably, patients who underwent ASCT exhibited significantly prolonged PFS compared to those who did not receive this consolidation strategy (Figure 5A,B). Patients who achieved CR or PR after induction therapy experienced significantly improved PFS following ASCT consolidation, irrespective of prior treatment with the R-MT regimen (Figures S2 and S3). To mitigate selection bias driven by differences in patient age and performance status, we compared ASCT recipients (n = 52) with clinically transplant-eligible patients who refused consolidation therapy (n = 18). Baseline characteristics between these two subgroups were confirmed to be statistically balanced (all *p* > 0.05, Table S5). Kaplan–Meier analysis confirmed that the ASCT group maintained significantly superior PFS compared to the refusal group (*p* < 0.01; Figure S6), suggesting that the survival benefit is attributable to the transplantation itself rather than superior baseline status.

Of the 10 cases who underwent consolidation treatment without attaining CR, 4 were assessed as having PR, 0 as SD, and 6 as PD after the conclusion of treatment. The majority of patients with PD succumbed at three months following ASCT (4/6, 67%). The efficacy of consolidation therapy was closely associated with PFS and OS. Cases not achieving CR upon consolidation therapy had median PFS and OS of 1.0 years and 2.2 years, respectively (Figure S5A,B).

To further identify treatment-related prognostic factors in PCNSL, Cox regression models were applied. Univariate analysis indicated that treatment regimen, depth of response after induction therapy, and ASCT were associated with PFS. Multivariate analysis confirmed all three as independent prognostic factors for PFS (Table 2). Specifically, consolidation with ASCT emerged as a robust independent predictor of improved PFS (HR = 0.416; 95% CI: 0.214–0.808; *p* = 0.010), corresponding to a 58.4% reduction in the risk of disease progression or death compared to patients who did not receive transplantation. Similarly, achieving a response (CR/PR) after induction therapy was associated with a 94.7% reduction in the risk of progression (HR = 0.053; 95% CI: 0.027–0.102; *p* < 0.001). Regarding overall survival, univariate analysis showed that depth of response and ASCT were associated with OS, while multivariate analysis further established depth of response as an independent prognostic factor for OS (Table S2). Although visual crossing of survival curves was observed in some univariate comparisons, the global test based on scaled Schoenfeld residuals for the multivariate Cox model indicated no significant violation of the proportional hazards assumption (*p* > 0.05). The discrepancies between univariate and multivariate results for PFS and OS may be attributable to subsequent salvage or maintenance therapies.

### 3.4. Treatment and Survival of Patients with Primary Refractory or Relapsed Disease

The salvage treatment following induction therapy exhibited a response rate of 69% (18/26), a median PFS of 4.2 months, and 1- and 2-year PFS rates of 66.6% and 53.2%, respectively. Of these cases, 31% (8/26) had radiation treatment, 27% (7/26) opted for BTK-containing regimens, and 23% (6/26) received MTX-containing regimens.

Of the 81 patients who achieved CR/CRu after induction therapy, 34 experienced relapse, which was predominantly confined to the CNS. Only two patients (6%) exhibited extra-CNS recurrence. Salvage therapy was administered to 27 patients, of whom 12 received MTX-based regimens and achieved a 2-year PFS of 56%, while 8 received BTK inhibitor-based regimens, with a corresponding 2-year PFS of 44%. Among patients treated with high-dose MTX salvage therapy, four subsequently underwent ASCT; none of these patients experienced disease progression during follow-up, which exceeded 2 years in all cases (maximum 8.6 years).

## 4. Discussion

PCNSL represents an immensely aggressive neoplastic disease with distinct biological traits, and the refinement of its therapeutic protocol continues to encounter significant obstacles. This study rigorously assessed the clinical efficacy of the entire therapy paradigm by a multicenter retrospective cohort analysis involving 140 PCNSL cases (median follow-up duration of 5.3 years), yielding three principal findings: (1) the R-MT regimen demonstrated remarkable safety and survival advantages during induction therapy, corroborated by a real-world cohort indicating that the induction regimen incorporating rituximab enhances patient survival; (2) ASCT consolidation therapy markedly improved prognosis; (3) patients exhibiting primary drug resistance and those failing to attain CR post-consolidation necessitate the prompt establishment of early intervention strategies.

The MT regimen demonstrates favorable safety in the induction therapy of PCNSL, yielding an ORR of 65%. The combination of rituximab (R-MT) markedly enhanced PFS (HR = 0.41, 95% CI 0.23–0.75; *p* = 0.004), corroborating previous rituximab-related studies suggesting a potential PFS benefit [13,18]. Despite the slightly inferior long-term survival of the R-MT regimen (7-year OS: 59.6%) compared to the MATRix regimen (7-year OS: 70%) [6]—presumably reflecting its relatively lower treatment intensity—its favorable low-toxicity profile (treatment discontinuation rate: only 9.2%) confers broader clinical applicability. Addressing the scarcity of large-scale Asian data, our study validates this “intermediate-intensity” approach as a pragmatic standard for real-world populations, particularly for elderly or unfit patients who may not tolerate the aggressive toxicity profile of MATRix-like regimens. In the future, MYD88/CD79B mutations may be utilized to identify individuals suitable for intense treatment [19,20], and targeted approaches, such as the use of combination BTK inhibitors, can be investigated to enhance efficacy [21,22].

Survival analysis indicated that ASCT was associated with a reduction in the risk of recurrence by 15.4% and 28.1% at 2 and 5 years, respectively (Figure 5A). The clinical relevance of our prognostic analysis is further underscored by the magnitude of the hazard ratios (HRs) observed in the multivariate model. Consolidation with ASCT demonstrated a robust protective effect (HR = 0.416), translating to a 58.4% reduction in the relative risk of disease progression or death compared to patients who did not receive transplantation. This substantial risk reduction confirms that the statistical significance of ASCT translates into a meaningful survival advantage in real-world practice, justifying its use as a standard of care for eligible patients. Further comparison between ASCT recipients and a specific control group of “clinically transplant-eligible but refusal” patients—confirmed to have balanced baseline characteristics (all *p* > 0.05, Table S5)—demonstrated that the survival benefit of ASCT derives from the treatment itself, rather than merely reflecting superior baseline performance status (*p* < 0.01, Figure S6). Patients who achieved CR after induction therapy and underwent consolidation treatment exhibited a 5-year PFS rate as high as 71.1% and a 5-year OS rate of 82.7% (Figure S5A,B). In contrast, patients with PR after induction therapy achieved a CR conversion rate of 75% following ASCT consolidation, with no statistically significant difference in survival outcomes compared to the post-induction CR group (*p* > 0.05, Figure S1A,B). This finding challenges the conventional paradigm restricting transplantation to CR achievers, suggesting that ASCT eligibility should be expanded to include fit PR responders to maximize long-term disease control. This finding may be attributed to the limitations of radiological assessment—residual lesions in certain PR patients may lack tumor metabolic activity, suggesting room for optimization of the current response assessment criteria. Furthermore, for patients achieving PR after induction therapy, proactive evaluation of the feasibility of ASCT consolidation is warranted, as this subset of patients may derive long-term survival benefits from consolidation treatment.

Patients with initial resistance (25%) and those who failed to achieve CR after consolidation (17%) had a dismal prognosis (median OS: 2.4 and 2.2 years, respectively). Mechanisms underlying resistance may involve impaired drug penetration across the blood–brain barrier (BBB), an immunosuppressive tumor microenvironment, and aberrant activation of the B cell receptor (BCR) signaling pathway [23,24,25].

The survival curve fails to reach a plateau, indicating an ongoing risk of relapse: the 2- and 5-year relapse rates were 41.5% and 67.6% in patients without consolidation, versus 26.1% and 39.5% in those with ASCT consolidation. Salvage regimens incorporating BTK inhibitors improved the 2-year PFS of relapsed patients to 44%, and all 4 patients who underwent salvage ASCT achieved sustained remission for ≥2 years, highlighting the potential of consolidation therapy. Novel strategies, such as dynamic monitoring of ctDNA methylation [26,27], PD-1 inhibitors combined with chemotherapy (ORR: 96.3% for the sintilimab + R-MT regimen) [28], and CAR-T therapy, offer new hope for relapsed patients [29,30,31]; nonetheless, their efficacy in PCNSL specifically requires further validation.

This study is inherently limited by its retrospective design, including selection bias (e.g., 50% of patients lacked cerebrospinal fluid cytology data) and inadequate evaluation of neurotoxicity. Future multicenter prospective studies are warranted to investigate the evolution of drug-resistant clones by integrating single-cell sequencing, and to establish a multimodal monitoring system incorporating ctDNA and PET-CT for dynamic risk stratification. Additionally, enhancing the assessment of neurocognitive function and quality of life will help advance the development of clinical precision medicine models.

## 5. Conclusions

This study confirms the central role of the R-MT induction regimen and ASCT consolidation in the management of PCNSL, and underscores the critical importance of dynamic assessment of response status for prognostic stratification. Significant unmet needs remain in the treatment of induction–refractory patients. Accordingly, future prospective studies and interdisciplinary collaboration are essential to further optimize risk-adapted strategies and facilitate the transition of PCNSL from a “refractory disease” to a “potentially curable” disease entity.

## Figures and Tables

**Table 1:** Baseline Patient Characteristics.

Characteristic			N (%)
Gender		Male	79 (56)
		Female	61 (44)
Age at diagnosis		<65	117 (84)
		≥65	23 (16)
IELSG		0–1	12 (9)
		2–3	50 (36)
		4–5	11 (8)
		NA	67 (48)
MSKCC		1	38 (27)
		2	38 (27)
		3	64 (46)
Ocular manifestations		Yes	33 (24)
		No	107 (76)
Meningeal involvement		Yes	6 (4)
		No	64 (46)
		Unknown	70 (50)
Biopsy technique		Stereotactic biopsy	93 (66)
		Surgery	47 (34)
Focality		Unifocal	69 (49)
		Multifocal	67 (48)
		Unknown	4 (3)
Induction regimen		MT	17 (12)
		R-MT	94 (67)
		Other	29 (21)
Consolidation	ASCT	Incorporating TT	19 (33)
		Other	29 (50)
		Unknown	4 (7)
	WBRT		6 (10)

Abbreviations: IELSG, International Extranodal Lymphoma Study Group; MSKCC, Memorial Sloan Kettering Cancer Center; MT, combination regimen of high-dose methotrexate and temozolomide; R-MT, combination regimen of rituximab, high-dose methotrexate and temozolomide; TT, Thiotepa; ASCT, Autologous Stem Cell Transplantation; WBRT, Whole-Brain Radiation Therapy.

**Table 2:** Univariate and multivariate analysis of clinical parameters on PFS.

Variables		Univariate			Multivariate		
		HR	95% CI	*p* Value	HR	95% CI	*p* Value
Gender							
	Male	Reference					
	Female	1.043	0.665–1.637	0.854			
Age (y)							
	≤65	Reference					
	>65	1.208	0.697–2.096	0.501			
MSKCC							
	1–2	Reference					
	3	0.999	0.641–1.556	0.997			
Deep structure involvement							
	No	Reference					
	Yes	0.925	0.579–1.478	0.745			
	Unknown	1.202	0.420–3.440	0.732			
Multiple lesions							
	Absent	Reference					
	Present	1.024	0.653–1.606	0.918			
	Unknown	1.638	0.505–5.318	0.411			
Regimen							
	MT	Reference			Reference		
	R-MT	0.411	0.225–0.749	**0.004**	0.519	0.279–0.967	**0.039**
	Other	0.831	0.415–1.663	0.601			
Response Depth to Induction Therapy							
	SD/PD	Reference			Reference		
	CR/PR	0.040	0.022–0.074	**<0.001**	0.053	0.027–0.102	**<0.001**
ASCT							
	No	Reference			Reference		
	Yes	0.216	0.119–0.392	**<0.001**	0.416	0.214–0.808	**0.010**

Factors with *p* < 0.1 in the univariate analysis were subjected to multivariate analysis afterwards. Forward stepwise Cox proportional-hazard modeling was used in multivariate analysis of risk factors. Bold values indicate statistical significance (*p* < 0.05). Abbreviations: MSKCC, Memorial Sloan Kettering Cancer Center; MT, combination regimen of high-dose methotrexate and temozolomide; R-MT, combination regimen of rituximab, high-dose methotrexate and temozolomide; SD, stable disease; PD, progressive disease; CR, complete response/unconfirmed complete response; PR, partial response; ASCT, Autologous Stem Cell Transplantation.

## Data Availability

Anonymized data are available from the corresponding author upon reasonable request from a qualified investigator.

## Supplementary Materials

The following supporting information can be downloaded at: https://www.mdpi.com/article/10.3390/cancers18030541/s1. Table S1. Transplant Conditioning Regimens and Patient Distribution. Table S2. Univariate and multivariate analysis of clinical parameters on OS. Table S3. Data Completeness for Key Baseline and Outcome Variables (N = 140). Table S4. Treatment-related adverse events during induction therapy (N = 140). Table S5. Baseline Characteristics Comparison Between ASCT Group and Transplant-Eligible Refusal Group. Figure S1. (A) Kaplan-Meier analysis of PFS stratified by induction therapy efficacy. (B) Kaplan-Meier analysis of OS stratified by induction therapy efficacy. Figure S2. (A) Kaplan-Meier analysis of PFS between ASCT-receiving and non-ASCT-receiving groups among patients with CR response to induction therapy. (B) Kaplan-Meier analysis of PFS between ASCT-receiving and non-ASCT-receiving groups among patients with PR response to induction therapy. Figure S3. (A) Kaplan-Meier analysis of PFS between ASCT-receiving and non-ASCT-receiving groups among patients achieving CR response to R-MT induction therapy. (B) Kaplan-Meier analysis of PFS between ASCT-receiving and non-ASCT-receiving groups among patients achieving PR response to R-MT induction therapy. Figure S4. (A) Kaplan-Meier analysis of PFS between <65 years and ≥65 years patient groups. (B) Kaplan-Meier analysis of OS between <65 years and ≥65 years patient groups. Figure S5. (A) Kaplan-Meier analysis of PFS stratified by consolidation therapy efficacy. (B) Kaplan-Meier analysis of OS stratified by consolidation therapy efficacy. Figure S6. Kaplan-Meier analysis of PFS stratified by consolidation decision (ASCT vs. Refusal) among transplant-eligible patients.
