# Supplementary material for: Clinical Characteristics and Prognosis of Primary Central Nervous System Lymphoma: A Retrospective Analysis
Source: Cancers (Basel). 2026 Feb 6;18(3):541. doi: 10.3390/cancers18030541 (PMC12896782; doi:10.3390/cancers18030541)
Supplement: Supplementary file 1 [file cancers-18-00541-s001.zip › cancers-4118476-supplementary.pdf]

cancers MDPI

Supplementary Materials: Clinical Characteristics and Prognosis of Primary Central Nervous System Lymphoma: A Retrospective Analysis

Shupeng Zhong, Linjun Zhao, Jin Chai, Lan Mi, Yan Xie, Lingyan Ping, Xiaopei Wang, Jun Zhu, Lijuan Deng and Yuqin Song

Table S1. Transplant Conditioning Regimens and Patient Distribution.

Conditioning Regimen	Number of Patients (n)	Percentage (%)
BCUN-TT	18	35
CBV	15	29
BEAC	7	13
BEAM	4	8
NA	4	8
FTM-TT	1	2
CFV	1	2
FEAC	1	2
TB	1	2

*Abbreviations: BCUN-TT, Carmustine - Thiotepa; CBV, Cyclophosphamide - Carmustine -Etoposide; BEAC, Carmustine - Etoposide - Cytarabine - Cyclophosphamide; BEAM, Carmustine - Etoposide - Cytarabine-Melphalan; NA, Not Available; FTM-TT, Fotemustine - Thiotepa; CFV, Cyclophosphamide – Fludarabine - Etoposide; FEAC, Fludarabine - Etoposide - Cytarabine - Cyclophosphamide; TB, Thiotepa - Busulfan.

Table S2. Univariate and multivariate analysis of clinical parameters on OS .

Variables	Univariate			Multivariate		
	HR	95% CI	*p* value	HR	95% CI	*p* value
Gender						
Male	Reference					
Female	1.223	0.709-2.111	0.469			
Age(y)						
≤65	Reference					
> 65	1.580	0.826-3.021	0.166			
MSKCC						
1-2	Reference					
3	1.246	0.720-2.158	0.431			
Deep structure involvement						
No	Reference					
Yes	1.372	0.745-2.524	0.310			
Unknown	1.518	0.438-5.269	0.511			
Multiple lesions						
Absent	Reference					
Present	1.498	0.856-2.622	0.157			
Unknown	1.989	0.465-8.497	0.353			
Regimen						
MT	Reference					
R-MT	0.637	0.317-1.279	0.205			

Other	0.867	0.381-1.973	0.733			
Response Depth to Induction Therapy						
SD/PD	Reference			Reference		
CR/PR	0.272	0.155-0.476	**< 0.001**	0.309	0.165-0.579	**< 0.001**
ASCT						
No	Reference			Reference		
Yes	0.458	0.235-0.892	**0.022**	0.730	0.347-1.537	0.407

*Factors with $p < 0.1$ in the univariate analysis were subjected to multivariate analysis afterwards. Forward stepwise Cox proportional-hazard modeling was used in multivariate analysis of risk factors. Abbreviations: MSKCC, Memorial Sloan Kettering Cancer Center; MT, combination regimen of high-dose methotrexate and temozolomide; R-MT, combination regimen of rituximab, high-dose methotrexate and temozolomide; SD, stable disease; PD, progressive disease; CR, complete response/unconfirmed complete response; PR, partial response; ASCT, Autologous Stem Cell Transplantation.

Table S3. Data Completeness for Key Baseline and Outcome Variables (N=140).

Variable	Available Data, n (%)	Missing Data, n (%)
Age	140 (100%)	0 (0%)
Sex	140 (100%)	0 (0%)
ECOG	140 (100%)	0 (0%)
MSKCC	140 (100%)	0 (0%)
Contrast-enhanced Brain MRI	140 (100%)	0 (0%)
PET/CT	95(68%)	45(32%)
Contrast-enhanced CT of the chest, abdomen, and pelvis	45(32%)	95(68%)
Bone Marrow Evaluation	140 (100%)	0 (0%)
Ophthalmologic Evaluation	140 (100%)	0 (0%)
MSKCC	140 (100%)	0 (0%)
IELSG	73 (52%)	67 (48%)
CSF Cytology	70 (50%)	70 (50%)
Neuropsychiatric Assessment	0 (0%)	140 (100%)
Response to Induction	140 (100%)	0 (0%)

*Abbreviations: ECOG, Eastern Cooperative Oncology Group; KPS, Karnofsky Performance Status; MSKCC, Memorial Sloan Kettering Cancer Center; IELSG, International Extranodal Lymphoma Study Group; CSF, Cerebrospinal Fluid.

Table S4. Treatment-related adverse events during induction therapy (N=140).

Adverse Events	All Grades, n (%)	Grade 3–4, n (%)
**Hematologic toxicities**		
Anemia	5(3.6%)	3(2.1%)
Neutropenia	19 (13.6%)	4(2.9%)
Thrombocytopenia	4 (2.9%)	2 (1.4%)
**Non-hematologic toxicities**		
Hepatotoxicity	22 (15.7%)	3 (2.1%)
Nephrotoxicity	15 (10.7%)	0 (0%)
Gastrointestinal disorders	14 (10.0%)	4(2.9%)
MTX elimination delay	13 (9.3%)	0 (0%)
Infection (including pneumonia)	10 (7.1%)	1 (0.7%)
Cardiovascular disorders	4(2.9%)	0 (0%)
Pulmonary toxicity	4 (2.9%)	0 (0%)
Neurotoxicity	2 (1.4%)	1 (0.7%)
Skin rash/Allergy	2(1.4%)	0 (0%)
**Treatment-related mortality**	0(0%)	-

Table S5. Baseline Characteristics Comparison Between ASCT Group and Transplant-Eligible Refusal Group.

Characteristic	ASCT Group (n=52)	Eligible Refusal Group (n=18)	p-value
**Sex**			0.796
Male	28	9	
Female	24	9	
**Age at diagnosis**			1.000
<65	51	18	
≥65	1	0	
**MSKCC**			0.315
1	19	3	
2	12	4	
3	21	11	
**Ocular manifestations**			0.106
Yes	13	1	
No	39	17	
**Response to Induction**			0.165
CR	40	17	
PR	12	1	

* P-values were calculated using Fisher's exact test.

* Abbreviations: MSKCC, Memorial Sloan Kettering Cancer Center; ASCT, Autologous Stem Cell Transplantation; CR, complete response; PR, partial response.

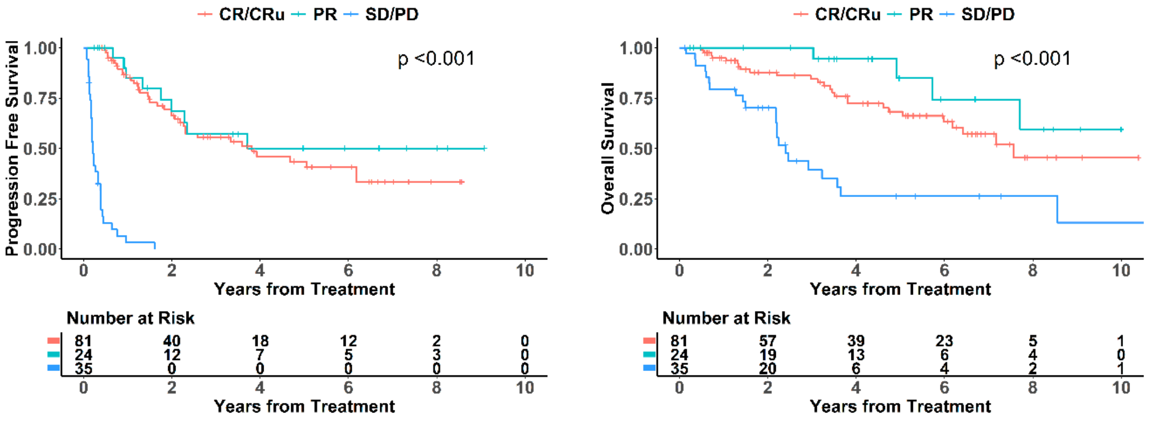

(A) (B)

Figure S1. (A) Kaplan-Meier analysis of PFS stratified by induction therapy efficacy. (B) Kaplan-Meier analysis of OS stratified by induction therapy efficacy.

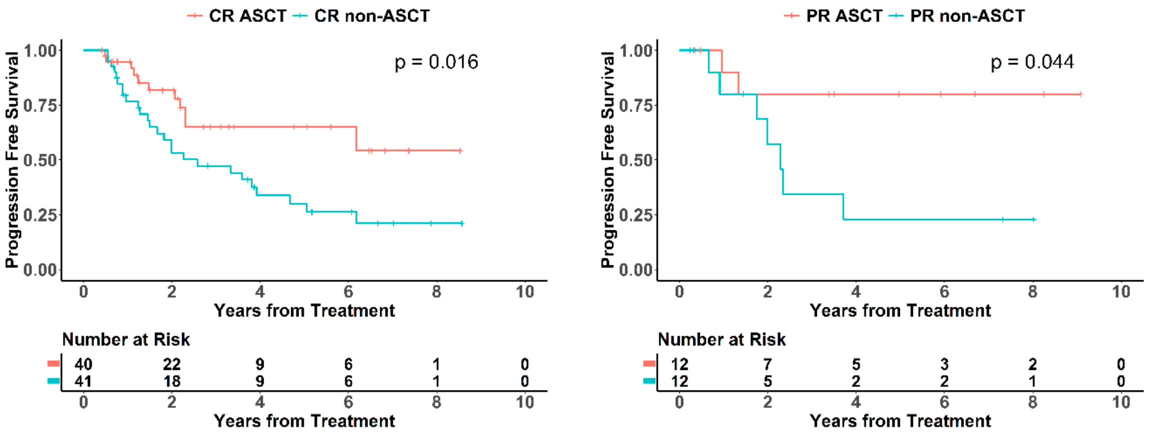

(A) (B)

Figure S2. (A) Kaplan-Meier analysis of PFS between ASCT-receiving and non-ASCT-receiving groups among patients with CR response to induction therapy. (B) Kaplan-Meier analysis of PFS between ASCT-receiving and non-ASCT-receiving groups among patients with PR response to induction therapy.

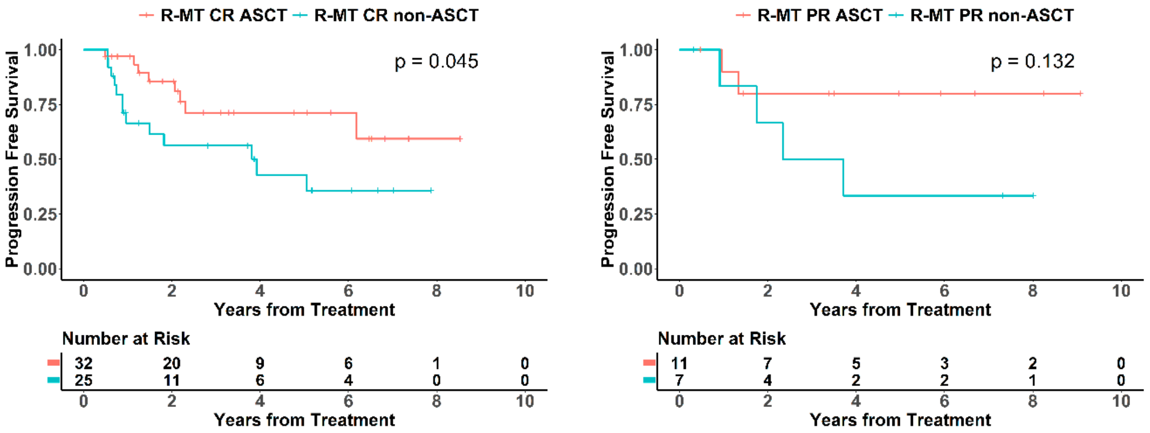

(A) (B)

Figure S3. (A) Kaplan-Meier analysis of PFS between ASCT-receiving and non-ASCT-receiving groups among patients achieving CR response to R-MT induction therapy. (B) Kaplan-Meier analysis of PFS between ASCT-receiving and non-ASCT-receiving groups among patients achieving PR response to R-MT induction therapy.

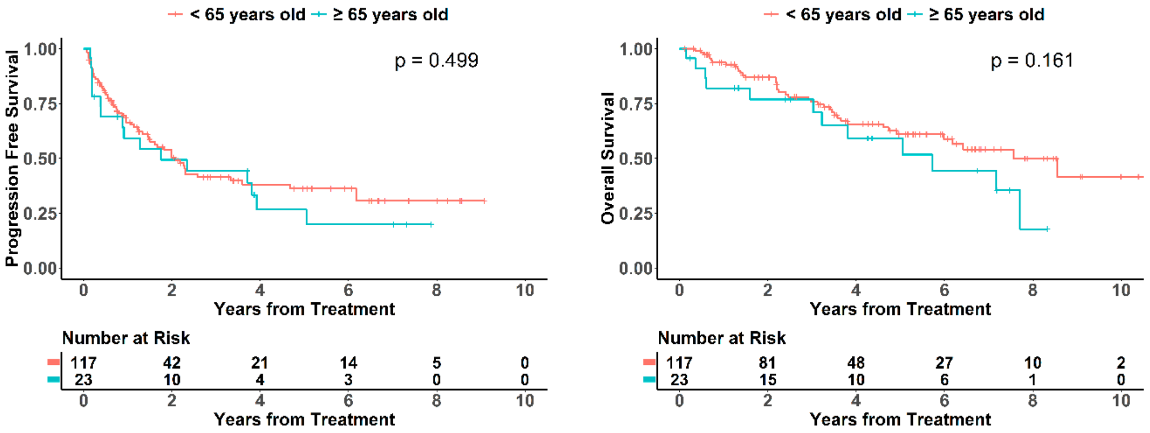

(A) (B)

Figure S4. (A) Kaplan-Meier analysis of PFS between <65 years and ≥65 years patient groups. (B) Kaplan-Meier analysis of OS between <65 years and ≥65 years patient groups.

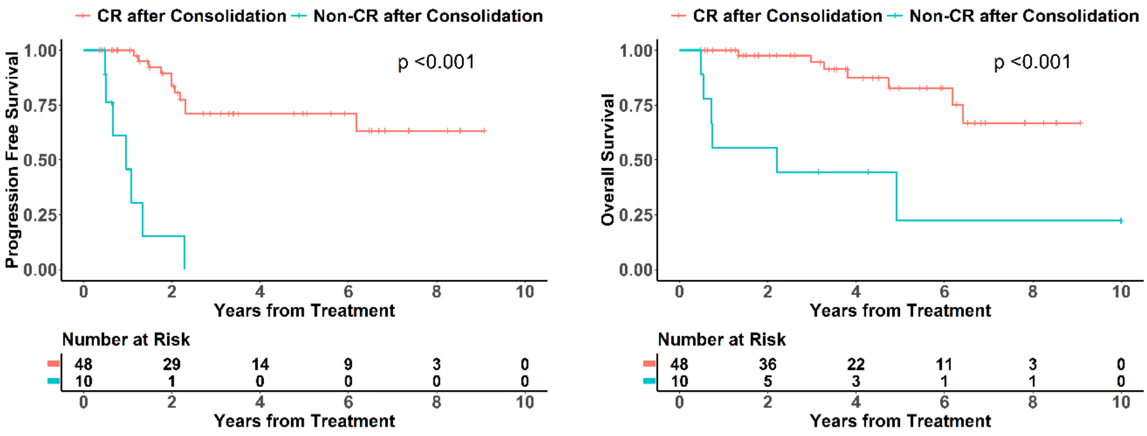

(A) (B)

Figure S5. (A) Kaplan-Meier analysis of PFS stratified by consolidation therapy efficacy. (B) Kaplan-Meier analysis of OS stratified by consolidation therapy efficacy.

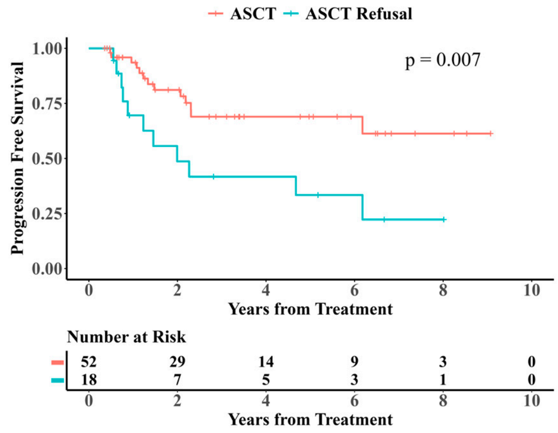

Figure S6. Kaplan-Meier analysis of PFS stratified by consolidation decision (ASCT vs. Refusal) among transplant-eligible patients.
